# The Treatment of Acute Intestinal Obstruction

**Published:** 1890-10

**Authors:** 


### Editorial.

### THE TREATMENT OF ACUTE INTESTINAL OBSTRUCTION.

The subject of the treatment of acute intestinal obstruction is one which possesses a vital interest for every practitioner of medcine, because it relates to a condition with which every physician is liable to meet at any time, and which, if not promptly remedied, will in the majority of cases prove fatal to the patient. As a result of the rapid progress of abdominal surgery in the last few years, intestinal obstruction has passed out of the domain of medicine and its treatment has fallen largely into the hands of the surgeon. The day is past when the medical attendant sat idly by, giving opium to relieve pain and waiting with folded hands for the almost inevitably fatal termination. The results of such treatment were simply appalling. To open the abdomen and remove the cause of obstruction is now regarded by all authorities as the only rational and satisfactory treatment. Such a course when undertaken with proper skill and at the proper time has produced results so immeasurably superior to the old plan of waiting for nature to accomplish that which she is seldom able to accomplish, that to neglect or postpone it is almost a surgical crime.

There are two points, however, in connection with this subject, which are so frequently overlooked that they cannot be too often or too strongly insisted upon. The success of the surgical treatment of intestinal obstruction depends upon a timely resort to operation and hence an early diagnosis. Unfortunately a positive diagnosis is not always possible in the early stages and hence the physician is tempted to rely upon therapeutic measures, which if not speedily successful are worse than useless.

Hoping against hope, he administers cathartics and enemata until the strength of the patient is exhausted, and when he awakens to the conviction that to surgery alone can he look for aid, the patient is moribund, and it is too late for an operation to avert the fatal termination.

As soon as symptoms of obstruction are recognized and have failed to yield readily to medical treatment, the time for surgical interference has arrived. Delay cannot strengthen the indication, but will materially lessen the chances of success. In the light of modern surgery, abdominal section is not a formidable procedure when performed upon a patient who is in good condition. The chances of death from delay are vastly greater than the chances of death from operation. The large mortality which has followed the operation in the past was due to procrastination, surgical treatment being resorted to only when the patient was *in extremis*. Statistics which seem to show that the chances are better for the patient without than with operative interference (those of Curschman, for instance) are valueless for this reason. To insure success the operation must be performed before the strength of the patient is exhausted and gangrene has occurred. This favorable period is usually limited to the first three days. But if a diagnosis can be made at the outset, the operation should be done immediately, since there is nothing to gain and much to lose by delay. In cases of undoubted intestinal obstruction, the chances of spontaneous recovery are practically nil. When there is doubt as to the diagnosis, the patient should be given the benefit of the doubt and the abdomen opened, a much safer procedure when undertaken early than waiting for time to solve the question.

A second point worthy of notice in connection with this subject is the futility of operating upon a dying patient. When undertaken as a last resort, the results are thoroughly discouraging. It is the reports of such cases that have thrown discredit upon the operation and caused surgeons to hesitate when surgical interference was strongly indicated. To operate for the sake of giving the patient "a last chance," is simply to delude ourselves with a hope which has no clinical basis. As has been well said, "The fact that a patient must die is no reason that the surgeon should become his executioner." It must be remembered that the indications to operate in any given case depend in the first place, upon the chance which the patient has of getting well without operation, and in the second place, upon the degree of probability with which success will follow the operation.

In conclusion we would quote the well-chosen words of Greig Smith upon the subject. He says: "The death-rate of laparotomy for intestinal obstruction is high, probably more than 70 per cent. Even thus the operation would still be justifiable; for nearly every case which recovers may be reckoned as saved from death, and the deaths are only hastenings of the natural termination. There can be no doubt that delay is the chief cause of mortality. We know how successful early herniotomy is; surely, in the face of recent exploits in abdominal surgery, early laparotomy for intestinal strangulation ought to be only a little less successful. Before abdominal distention has come on, before the bowel has become inflamed, and before the patient's strength is exhausted, I have no hesitation in affirming that, in competent hands, laparotomy for intestinal obstruction would not have a mortality above fifteen per cent."

